# Biological Effects and Mechanisms of Caspases in Early Brain Injury after Subarachnoid Hemorrhage

**DOI:** 10.1155/2022/3345637

**Published:** 2022-07-05

**Authors:** Yiwen Wu, Yuchun Liu, Chenhui Zhou, Yuefei Wu, Jie Sun, Xiang Gao, Yi Huang

**Affiliations:** ^1^Department of Neurosurgery, Ningbo First Hospital, Ningbo Hospital, Zhejiang University School of Medicine, Ningbo, Zhejiang 315010, China; ^2^Department of Neurology, Ningbo First Hospital, Ningbo Hospital, Zhejiang University School of Medicine, Ningbo, Zhejiang 315010, China; ^3^Key Laboratory of Precision Medicine for Atherosclerotic Diseases of Zhejiang Province, Ningbo, Zhejiang 315010, China

## Abstract

Caspases are an evolutionarily conserved family of proteases responsible for mediating and initiating cell death signals. In the past, the dysregulated activation of caspases was reported to play diverse but equally essential roles in neurodegenerative diseases, such as brain injury and neuroinflammatory diseases. A subarachnoid hemorrhage (SAH) is a traumatic event that is either immediately lethal or induces a high risk of stroke and neurological deficits. Currently, the prognosis of SAH after treatment is not ideal. Early brain injury (EBI) is considered one of the main factors contributing to the poor prognosis of SAH. The mechanisms of EBI are complex and associated with oxidative stress, neuroinflammation, blood-brain barrier disruption, and cell death. Based on mounting evidence, caspases are involved in neuronal apoptosis or death, endothelial cell apoptosis, and increased inflammatory cytokine-induced by apoptosis, pyroptosis, and necroptosis in the initial stages after SAH. Caspases can simultaneously mediate multiple death modes and regulate each other. Caspase inhibitors (including XIAP, VX-765, and Z-VAD-FMK) play an essential role in ameliorating EBI after SAH. In this review, we explore the related pathways mediated by caspases and their reciprocal regulation patterns after SAH. Furthermore, we focus on the extensive crosstalk of caspases as a potential area of research on therapeutic strategies for treating EBI after SAH.

## 1. Introduction

Caspases are a large family of evolutionarily conserved proteins that finely regulate the process of cell death. Apoptotic and inflammatory caspases mediate apoptosis and pyroptosis signaling pathways, respectively [1]. The dysregulation of caspase activation is a feature of many diseases (inflammatory, communicable, metabolic, malignant, and neurodegenerative) and is indicated as the pathogenic mechanism in autoinflammation, autoimmunity, and tumorigenesis [2]. Caspase has been implicated in brain injury, as well as neurodegenerative, neuroinflammatory [3], and cerebrovascular diseases.

Subarachnoid hemorrhage (SAH) can cause stroke and neurological damage and is often lethal [4, 5]. Cerebral vasospasm (CVS) after SAH is the cause of many damaging effects. Accordingly, many studies have focused on this sequela of SAH [6]. Although widely recognized to be associated with SAH, the clinical treatment of CVS has not improved significantly. Based on recent laboratory evidence, early brain injury (EBI) caused by SAH leads to the occurrence and development of pathology within 72 h of the initial hemorrhage. As a result, the research focus has recently shifted from CVS to EBI [7, 8].

EBI is currently considered a crucial factor in the poor prognosis of SAH [9, 10]. Neuroinflammation, oxidative stress, and blood-brain barrier (BBB) disruption are the mechanisms and pathological changes associated with EBI. Brain edema and cell death have also been observed in this condition [11–14]. The response of caspases includes various apoptotic and inflammatory factors, which are remarkably important in EBI after SAH. Therefore, we aimed to reveal the clinical research status of caspase-mediated EBI after SAH, focusing on the relationship between these biomarkers and EBI. We intended to discuss the possible cellular and molecular mechanisms related to these biomarkers and their clinical significance and review potential therapeutics for EBI based on the targeting of caspases.

## 2. Pathways Associated with Caspase Activation in EBI after SAH

**Figure 1:**
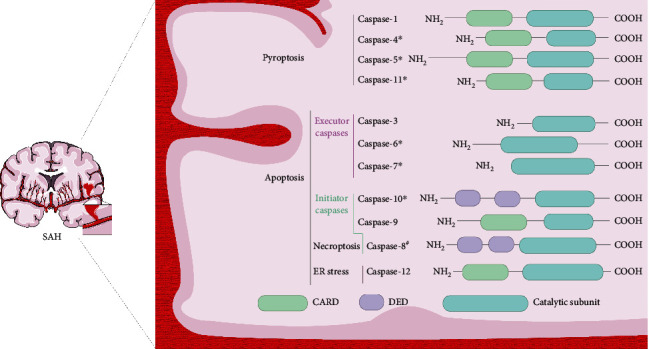
Functional classification and domain architecture of caspases in SAH. Caspases are classified in SAH as pyroptotic or apoptotic caspases according to their function and domain structure. Apoptotic caspases can be further subdivided into initiator caspases and executioner caspases. The function of initiating caspases is to initiate apoptosis by activating executioner caspases. Furthermore, necroptosis occurs when caspase-8 is inhibited or knocked out. Caspase-12 is structurally like inflammatory caspases but may be activated by endoplasmic reticulum stress to participate in apoptosis. ^∗^Until today, caspase-4, caspase-5, caspase-6, caspase-7, caspase-10, and caspase-11 have not been experimentally demonstrated in SAH, and their roles in SAH remain undefined. ^#^Inhibition of caspase-8 activates necroptosis.

“Caspase” is the abbreviated form of cysteine-dependent asparagine-specific protease [15]. Members 1–14 of the caspase family were designated based on the order of their discovery [16]. Upstream initiator and downstream effector caspases are classified into families according to their domain architecture and functions (Figure 1) [17]. The protease domains of caspases consist of an amino-terminal and catalytic subunit of different sizes [17]. Initiator caspases induce the activation and recruitment of other caspases in multiprotein complexes through their amino-terminal domains, called caspase recruitment domains (CARDs: caspase-1, caspase-2, caspase-4, caspase-5, caspase-9, and caspase-11) or death effector domains (DED: caspase-8 and caspase-10) [18, 19]. The initiator caspase is activated by dimerization when combined with an activation platform [19]. Downstream executioner caspases (caspase-3, caspase-6, and caspase-7) are then initiated by proteolytic cleavage of the initiator caspases. This signal transduction, from initiation by upstream caspases to downstream caspases, is necessary for the function of cysteine proteases [20].

Similar to their role in other stroke-related diseases, caspases participate in EBI regulation by inducing apoptosis. Apoptosis occurs via two approaches: endogenous apoptosis, which occurs in and is mediated by the mitochondria, and exogenous apoptosis, which occurs outside the mitochondria and is induced by death receptors called the exogenous apoptotic cell death pathway [21, 22]. The expression of caspase-1 leads to the induction of pathways that result in a type of regulated cell death (RCD), distinct from apoptosis, called pyroptosis, which is associated with the influence of inflammatory cytokines [23]. Caspase-1, caspase-4, caspase-5, and caspase-11 are collectively referred to as inflammatory caspases owing to their functions in these processes. The activation of the caspase-1-mediated pyroptosis pathway is of great significance in regulating EBI.

Apoptotic caspases can be divided into initiator caspases that receive upstream apoptosis initiation signals and effector caspases that ultimately execute apoptosis. The initiator caspases, -8, -9, and -10, initiate apoptosis by activating the executor caspases, -3, -6, and -7. Caspase-2 is involved in the cell cycle [24]. Although mechanistically obscure, caspase-14 appears to have a highly specific role in other tissues, such as the skin [25].

## 3. Intrinsic Mechanisms of Classic Apoptosis

**Figure 2:**
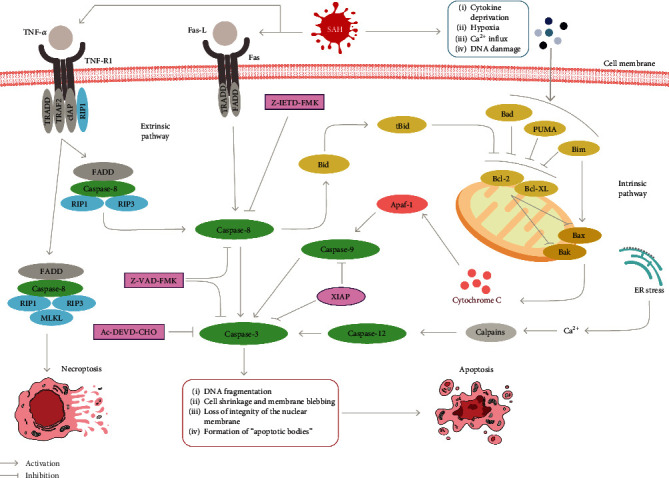
Apoptosis and necroptosis in SAH. The intrinsic pathway is activated by various stresses after SAH prompting activation of Bak and Bax and release of cytochrome c, which binds to Apaf-1 and then activates through self-cleavage of procaspase-9, further initiating the apoptotic cascade of caspase-3. The extrinsic pathway is activated through membrane receptor formation complexes, and the programmed death cascade induced through caspase-8 to caspase-3 activation. In addition, endoplasmic reticulum stress activates caspase-12 via calcium-activated calpains and ultimately acts on caspase-3 to mediate apoptosis. The natural inhibitor XIAP, the broad-spectrum inhibitor Z-VAD-FMK, the artificial inhibitors Z-IETD-FMK and Ac-DEVD-CHO, which exert inhibitory functions on caspase-8, caspase-9, and caspase-3, respectively, can effectively alleviate apoptosis in EBI. Necroptosis is triggered downstream of the death domain receptor TNFR. Upon detection of a “death signal,” RIP1 is activated and the subsequent formation of a RIP homotypic interaction motif (RHIM) begins to recruit RIP3. The RIPK1/RIP3 complex recruits and phosphorylates MLKL to form necrosomes. Ultimately, MLKL acts on the cell membrane to form pores that lead to necroptosis by allowing the uncontrolled release of intracellular substances.

The caspase-dependent intrinsic pathway is related to the highly regulated activation of the mitochondrion (Figure 2). Responses generated by multiple stressors inhibit Bcl-2 and Bcl-XL. They subsequently activate Bak and Bax, which are proapoptotic proteins, ultimately increasing the permeability of the mitochondrial outer membrane [26]. This change results in the loss of mitochondrial proteins, such as cytochrome c, which allows the efflux of proteins to the cytoplasm, leading to the formation of a complex with apoptotic protease activator 1 (Apaf-1). This complex is involved in the activation of caspase-9, which leads to the processing and activation of effector caspases [27].

Apoptosis in EBI after SAH is mainly related to the activation of caspase-3; however, data related to caspase-7 are limited. “Executioner caspases” induce apoptosis-related morphological changes in cells, including chromosomal DNA fragmentation, cell shrinkage, and the formation of “apoptotic bodies” [28]. According to various studies, caspase-dependent intrinsic pathways are activated early after SAH [29, 30]. After SAH, a high level of caspase-3 is also observed in serum, which is closely related to time [31, 32].

Some pathways activated by SAH may directly regulate caspase-mediated apoptosis, mainly via the phosphorylation of pro- and antiapoptotic proteins (Bad, Bax, Bcl-2, and Bcl-xL) [33]. Akt is one of the most studied proteins in EBI following SAH. Akt is a serine/threonine-specific protein kinase and a critical antiapoptotic signaling molecule downstream of phosphoinositide 3-kinase (PI3K) in the signaling cascade. PI3K activation regulates cell growth, cell cycle entry, and survival [34]. In fact, PI3K activates Akt, which then regulates the inactivation of many substrates (including Bad [35], fork head transcription factor [36], c-Raf [37], and caspase-9 [35]) to promote cell survival. PI3K and Akt can inhibit apoptosis. Akt phosphorylates cyclic AMP response element-binding protein (CREB), which promotes the expression of Bcl-2 [38]. I-*κ*B kinase (IKK) phosphorylation is activated by Akt, leading to I-*κ*B*α* degradation through the activation of the ubiquitin/protease system, which can activate NF-*κ*B for its entrance into the nucleus and restore its activity. NF-*κ*B plays various roles in mediating apoptosis and cell proliferation by regulating related genes, such as Bcl-2 family members and inhibitors of apoptosis protein (IAP) [39]. Activation of the PI3K/Akt pathway can alleviate neuronal apoptosis in the rat model of SAH and play a fundamental role in EBI [40, 41]. Downregulation of the PI3K pathway in SAH can reduce the protein levels of Bax and cleaved-caspase-3 [42]. The timing of Akt phosphorylation in different brain regions differs after SAH. Akt phosphorylation takes 24 h in the hippocampus but is rapid in the cerebral cortex [43]. Such findings may be clinically significant for EBI therapy after SAH [32].

In addition to mediating apoptosis and serving as the key molecule in the apoptotic pathway, caspase-3 may also be a prognostic marker for disease progression following SAH. Caspase-3 activity has been found to correlate with SAH severity, possibly reflecting the extent of neuronal damage [32]. Caspase-3 was also associated with clinical outcomes six months after SAH. The production of cleavage products involved in apoptosis has been demonstrated to be a prognostic marker after SAH. During apoptosis, caspases cleave cytokeratin-18 (CK-18), which generates fragments called caspase-cleaved CK- (CCCK-) 18 that are subsequently released into the blood. High CCCK-18 expression levels positively correlate with poor prognosis and higher expected mortality in patients with SAH [44]. Apoptosis-inducing caspases can hydrolyze cytoskeleton axon protein *α*-II from a molecular weight of 120–280 kDa, called SBDP120. Caspase-mediated apoptosis of SBDP120 was observed on day three post-SAH, with increasing concentrations until day seven [45]. Caspase-3 and its cleavage products also demonstrated excellent predictive functions as prognostic disease markers in EBI. More molecules related to these processes are expected to be discovered and recognized to be of diagnostic and prognostic value, enabling timely and relevant clinical interventions.

## 4. Extrinsic Mechanisms of Classic Apoptosis

The death receptor signaling pathway is a mode of apoptosis independent of the mitochondrial signaling pathway (Figure 2) [46]. To date, extracellular signaling ligands, such as Fas-L and cytokine tumor necrosis factor-*α* (TNF-*α*), are involved in the Fas and tumor necrosis factor receptor- (TNFR-) mediated death receptor pathway [47]. After the TNF ligand is connected to its receptor (TNFR1), a complex comprising multiple molecules (tumor necrosis factor receptor-associated factor (TRAF), TNF receptor type 1-associated death domain (TRADD), receptor-interacting protein kinase 1 (RIP1), and IAP) recruits E3 ligase, causing the inhibitor of apoptosis (cIAP) to ubiquitinate RIP1 to maintain cell stability. Notably, an imbalance in this pathway mediates the activation of caspase-8. After the Fas-L is connected to Fas, specific adaptive proteins, such as TRADD and Fas-associated death protein (FADD), form a death-initiation signaling complex (DISC) in the cytoplasm, which initiates the caspase-8 activation cascade. Activated caspase-8 can cleave the Bcl-2 interacting domain (BID) into a truncated active form (tBid), affecting caspase-3 by participating in mitochondria-mediated endogenous apoptosis. Collectively, the above pathways can ultimately induce the apoptotic pathway by activating caspase-3 [1, 23].

Transmembrane signaling by activating the TNFR and Fas family might be the mechanism underlying death receptor pathway-mediated apoptosis following SAH. Endothelial cells play a vital role in maintaining the function and integrity of the BBB. Disruption of the BBB ultimately aggravates the consequences of SAH. Apoptotic death of endothelial cells is one of the mechanisms whereby such aggravation occurs [48]. According to previous studies, death receptor signaling in SAH can lead to endothelial cell apoptosis after SAH. Further, this pathway is at least partially mediated by TNF*α*-receptor-1 and recruits and activates caspase-8, ultimately affecting endothelial cell damage, characterized by caspase-3-mediated apoptosis [30, 49]. The expression levels of these cytokines may be increased in response to stress and cause apoptosis of endothelial cells through endocrine or paracrine mechanisms. The immune response may also occur through hematopoietic cells, leading to the activation of death receptors [50]. Therefore, an external mechanism of apoptosis may contribute to late cell death after SAH; however, its role in mediating early cell death in EBI requires further investigation. Notably, relevant research is currently limited.

According to emerging evidence, hemolysate induces the upregulation of caspase-8 expression levels in SAH, suggesting that death receptor-related pathways are involved in hemolytic substance-induced apoptosis in cortical neurons [51]. Caspase-8 and FADD are associated with the nucleotide-binding oligomerization domain, leucine-rich repeat, and pyrin domain containing 3 (NLRP3) inflammasome, resulting in gasdermin D- (GSDMD-) mediated cell death in the form of pyroptosis [1, 52]. Owing to these findings, we opted to focus on the early functions of exogenous apoptosis in SAH. Of note, the actual mechanism underlying the pathway affecting EBI may markedly differ from the known mechanism.

## 5. Endoplasmic Reticulum Stress-Induced Apoptosis

Caspase-12 may regulate apoptosis through a new mechanism during the pathophysiological process of SAH [53]. Caspase-12 is mainly produced in the endoplasmic reticulum (ER) of nerve cells and is the main molecular marker of ER stress-mediated cell death [53]. The ER is a unique organelle with considerable importance in cell survival and homeostasis. Caspase-12 knockout mice are insensitive to other apoptotic pathways but are significantly resistant to ER stress-induced apoptosis, indicating that caspase-12 is a specific ER stress-induced apoptosis regulator [54].

Currently, the apoptotic role of caspase-12 in the ER remains controversial. When the intracellular calcium concentration is pathologically increased during ER stress, calcium-activated proteases (calpains) are activated (Figure 2), leading to the initiation of caspase-12 activation. Another theory suggests that caspase-7 and caspase-12 can form a complex with glucose-regulated protein 78 (GRP78) on the surface of the ER. This interaction leads to the cleavage of caspase-12, mediated by caspase-7, and finally the activation of caspase-3 [55]. Nevertheless, the specific mechanism by which caspase-7 is recruited to the ER and activated during ER stress has not been clearly elucidated. Caspase-12 may cause apoptosis through autocatalytic processing during ER stress [54]. In C2C12 cell-line myoblasts, caspase-12 activates procaspase-9, which subsequently cleaves caspase-3 but is not associated with the Apaf-1/cytochrome c pathway [56]. In immortalized mouse embryonic fibroblasts (Sak2), caspase-12 was found to induce the activation of procaspase-9 and the processing of caspase-3, but independently of Apaf-1 [57]. Other studies strongly suggest that caspase-12 has insufficient catalytic activity compared to that of other caspases and is limited to self-processing [58, 59].

Based on relevant experiments, ER stress can be induced and reaches its peak 24 h after SAH. Further, the inhibition of ER stress aggravates neurological deficit (in the SAH rat model), indicating that ER stress has a potentially protective function against EBI after SAH [60]. Li et al. [53] reported that the concentration of caspase-12 peaked at 72 h after SAH, which is substantially later than the onset of ER stress in EBI, and continued to increase until three days after SAH. The activation of caspase-12 may be related to long-term apoptosis caused by SAH and the continuous and cumulative effect of ER stress [53]. Through experiments, apoptosis of rat hippocampal neurons was induced by ER stress in SAH [61]. Therefore, the apoptotic pathway mediated by caspase-12 offers promising therapeutic prospects for SAH treatment. Further studies could be conducted using caspase-12-specific inhibitors to identify the role of caspase-12 in SAH mediation, including apoptosis.

## 6. Necroptosis in EBI after SAH

Necroptosis is a complex RCD mechanism triggered by TNF-*α* activation and mediated by activated RIP1 [62]. Necroptosis is essentially a nonapoptotic mode of death that clears pathogens and promotes tissue repair [63]. Necroptosis usually occurs after the inhibition of caspase-8, and its pathway remains intact during infection or when oncogenic mutations result in the loss of caspase activation (Figure 2). Therefore, necroptosis is considered a protective switch-like mechanism [64, 65]. The specific process of necroptosis involves the activation of RIP1 by TNF-*α*/TNFR1 and the subsequent recruitment of RIP3 through the RIP homotypic interaction motif (RHIM). The interaction of these two proteins results in the phosphorylation of RIP3, which activates its kinase in proper sequence, resulting in increased phosphorylation of RIP3 and downstream mixed lineage kinase domain-like pseudokinase (MLKL). The interaction of RIP1-RIP3 and RIP3-MLKL leads to the formation of a necrosome complex to execute necroptosis [66, 67]. Yuan et al. [68] demonstrated that the necrosome complex was formed by RIP3, RIP1, and MLKL in the brain tissue after SAH, and RIP3 peaked at 24 h after SAH. In the experiment, relevant inhibitors were used to demonstrate that increased RIP3 levels aggravate brain edema and disrupt the BBB, indicating that necroptosis is also crucial in EBI. Yang et al. [69] revealed that RIP1 and RIP3 levels are notably elevated in the hippocampus after SAH. In addition, compared to the untreated SAH group, the injection of a specific inhibitor (necrostatin-1) during necroptosis reduced brain edema and neuronal damage and improved synaptic structure. In a mouse model of SAH, necrostatin-1 was demonstrated to reduce RIP3 and MKLK protein levels and endothelial cell death and protect the BBB from EBI. Such finding provides new insights into the targets of EBI therapy [70].

## 7. Caspase-1-Mediated Pyroptosis Pathway

The activation of inflammatory caspases leads to a cell-lytic type of RCD called pyroptosis. This proinflammatory RCD has been noted to be distinct from apoptosis [71, 72]. Apoptosis is generally considered nonlytic and removes unwanted cells; however, pyroptosis is a distinct form of death involved in lysis and cell membrane disruption [73]. The primary role of pyroptosis is to clear cells affected by pathogen-associated molecular pattern (PAMP) or damage-associated molecular pattern (DAMP). This primary effect is achieved by facilitating the recruitment of effector cells, such as monocytes, to the injury site. Pyroptosis is mediated by inflammasomes, such as absent in melanoma 2- (AIM2-) like receptor (ALR), TLR, retinoic acid-inducible gene I-like receptor, and a receptor located in the cytoplasm called NLR [74]. NF-*κ*B receives signals from these receptors to stimulate the expression of certain cytokines, whereas others signal caspase-1 to induce pyroptosis [75]. Nonetheless, among the different inflammasomes found in the central nervous system (CNS), only a few, namely, AIM2, NLRP1, NLRP2, and NLRP3, have been studied. Among these inflammasomes, the most common experimental studies related to the NLRP3 inflammatory body. Based on increasing evidence, these receptors play a crucial role in EBI, vasospasm, and delayed deterioration of neurological function after SAH [76]. These receptors have recently been shown to be critical players in EBI after SAH [77].

**Figure 3:**
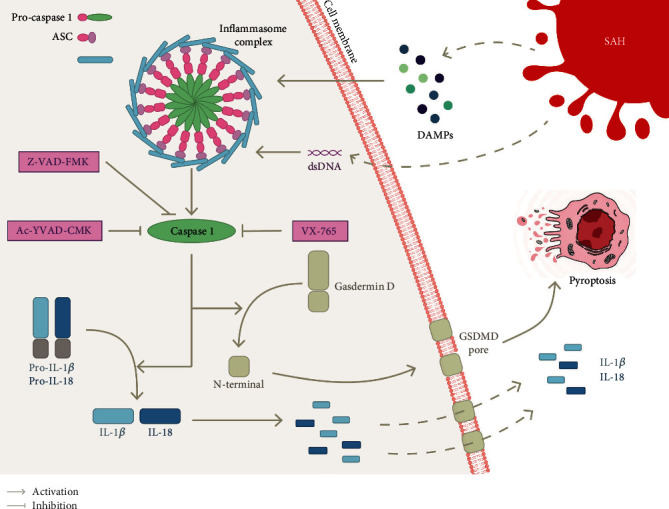
Pyroptosis pathway in SAH. Pyroptosis is an inflammatory form of cell death that is stimulated in SAH by intracellular sensor proteins such as NLRP3 (used as an example here) or AIM2 that detect damage-associated molecular patterns (DAMPs) or cytoplasmic double-stranded DNA (dsDNA). Upon receiving these stimuli, these sensors recruit an adaptor, the apoptosis-related speck-like protein ASC, which in turn recruit procaspase-1 to form the inflammasome complex and activate caspase-1 within it. The activation of caspase-1 cleaves precursors of the interleukin-1 family cytokines IL-1*β* and IL-18. In addition, caspase-1 also activates gasdermin D (GSDMD), causing its N-terminal domain to be cleaved and forming a pore (GSDMD pore) in the plasma membrane. Mature IL-1*β* and IL18 are released along the GSDMD pore and cause cell swelling eventually triggering pyroptosis. Moreover, both the broad-spectrum caspase inhibitor Z-VAD-FMK and caspase-1-specific inhibitors (VX-765 and Ac-YVAD-CMK) can alleviate EBI after SAH by inhibiting the caspase-1-mediated pyroptosis pathway.

As shown in Figure 3, the inflammasome is a multimolecular complex composed of sensor proteins (such as NLRP3), adaptor proteins, (namely, apoptosis-related speck-like protein ASC, which includes PYRIN domains (PYDs) and CARDs), and effector protein procaspase-1 [78, 79]. This complex activates caspase-1, which in turn induces GSDMD. Further, this process triggers the development and excretion of the proinflammatory cytokines IL-1*β* and IL-18. GSDMD is the substrate of caspases that is responsible for the formation of membrane pores. The N-terminal fragments generated after the cleavage of GSDMD form pores in the plasma membrane [80]. Further, the damaged cell membrane cannot regulate the fluid balance inside and outside the cell, which causes rapid swelling and rupture of the cell, eventually inducing pyroptosis. The processing of these cytokines is closely associated with GSDMD cleavage-driven cell lysis. However, some controversy prevails regarding this notion. According to some reports, the secretion of cytokines during pyroptosis may not be related to cell death [81, 82]. Pyroptosis was given a different definition in some related studies, as RCD caused by pyroptosis was proposed to be mediated by GSDMD and not by caspase-1 [80]. This discrepancy may be caused by the definition of cell death and membrane permeability. Nevertheless, the mechanism of pyroptosis requires further investigation. Another pyroptosis pathway can lead to the activation of caspase-11, caspase-4, and caspase-5 through lipopolysaccharides and ultimately affect GSDMD [83]. However, limited studies have been performed on the atypical pyroptosis pathway in SAH, which are not discussed in this review.

After SAH, blood and blood products that accumulate in the subarachnoid space form the inflammasome complex, enhance neuroinflammation, and activate caspase-1, which may worsen patient prognosis [84]. According to recent evidence, caspase-1 activation induces pyroptosis, leading to the release of the inflammatory cytokines IL-1*β* and IL-18, which results in poor outcomes in EBI after SAH [85]. Clinical experiments have shown that the level of caspase-1 peaked 24 h after SAH and was accompanied by an increase in necrotic nerve cells, most of which were neurons [86].

NLRP3-mediated neuroinflammation and neuronal pyroptosis have been verified to play vital roles in SAH. NLRP3 can induce EBI after SAH by inducing the overexpression of inflammatory cytokines through the activation of proinflammatory cytokines [87–89]. Inhibition of the NLRP3 inflammasome by hydrogen-rich saline [90], melatonin [91], minocycline [87], and atorvastatin [92] can alleviate EBI after SAH. Similarly, AIM2 inflammasome-mediated GSDMD-induced pyroptosis has been demonstrated to be associated with EBI after SAH. Furthermore, the more severe the SAH, the higher the expression level of AIM2 in the cerebrospinal fluid [92]. Previously, Krajewska et al. [93] revealed that the inhibition of caspase-8 after SAH could improve long-term neurological function. In other diseases, caspase-8 has been demonstrated to regulate NLRP3 and promote IL-1*β* [94]. Subsequent experiments have shown that caspase-8 can participate in caspase-1-mediated pyroptosis through NLRP3, improving the nervous system by reducing neuroinflammation [52]. Such finding suggests crosstalk between caspase-mediated pyroptosis and apoptosis in SAH. Additional extensive research will be discussed in subsequent sections.

## 8. Signal Crosstalk in Pyroptosis, Apoptosis, and Necroptosis

**Figure 4:**
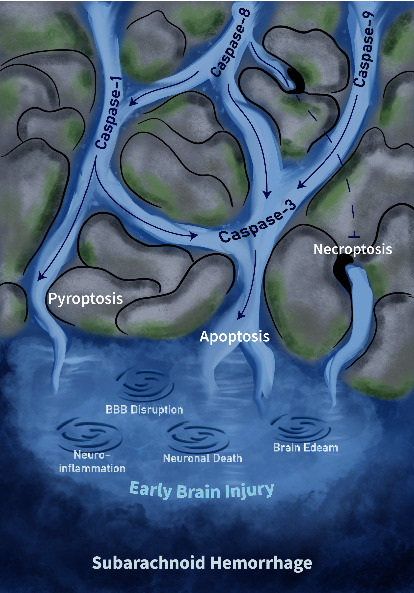
Relationship between caspase-mediated programmed cell death's crosstalk and the underlying pathological mechanisms in EBI.

Although apoptosis, pyroptosis, and necroptosis have long been described as unique and independent cell death pathways, a broad interplay between these pathways is increasingly being found (Figure 4). Prior experiments revealed an interaction between pyroptosis and apoptosis, as caspase-7 was identified as a potential substrate for caspase-1 in macrophages during its first discovery in 2008 [95]. Inflammasome and caspase-1 activation can lead to cleavage of the apoptotic substrate, poly (ADP-ribose) polymerase 1 (PARP1) [96]. Changes in GSDMD concentrations, which might cause caspase-1 to play distinct roles in cell death, were discovered in subsequent studies.

The figure depicts the streams of caspase-mediated RCDs' interactions and the pathological mechanisms that ultimately lead to EBI by them. Caspase-1 is involved in the mediation of the pyroptotic pathway but is regulated upstream by caspase-8 as well. Caspase-9 and caspase-8 finally activate the caspase-3-mediated apoptosis pathway through endogenous and exogenous pathways, while caspase-1 can also participate in apoptosis by regulating caspase-3 under specific circumstances. In the state where caspase-8 is inhibited, necroptosis is activated. The swirls represent EBI-related pathological processes triggered by RCDs including neuroinflammation, disruption of the blood-brain barrier, neuronal death, and brain edema.

In the absence of GSDMD, caspase-1 can initiate apoptosis through BID-dependent pathways and ultimately activate caspase-3 [97]. The role of BIDs in caspase-1-induced apoptosis is critical. Some inflammasomes can activate apoptosis independently of caspase-1 [98]. In cells, such as neurons and mast cells, with low or no GSDMD expression, this type of regulation is of physiological importance. Caspase-3 was found to cleave GSDMD at the D87 site in monocytes and macrophages and pyroptosis, originally mediated by caspase-1, was not activated after cleavage [98]. Thus, not only are caspase-3 and caspase-7 unable to trigger pyroptosis-mediated cell death by cleaving or activating pyroptosis-associated caspase [99], but they also inactivate GSDMD, a key substrate for pyroptosis, by affecting cell lysis. Activation of caspase-3 and caspase-7 follows the lysis of GSDMD and the onset of pyroptosis. This process is slower than that of pyroptosis and may be a fine-tuned regulated negative feedback mechanism that must be verified. GSDME is cleaved by caspase-3 and can induce pyroptosis upon activation, as observed in the family of GSDMs; however, this mode may be activated by specific molecules, such as chemotherapeutic drugs [100]. Recent experiments revealed that the knockout of caspase-1 in SAH not only alleviates neuronal pyroptosis but also reduces neuronal apoptosis induced by caspase-3 activation. However, caspase-3 expression is not affected by caspase-1 knockdown [101].

Caspase-8 is another critical link between pyroptosis and apoptosis. Mechanistically, caspase-8 and FADD can activate caspase-1-dependent pyroptosis [102]. Furthermore, inhibition of transforming growth factor beta-activated kinase 1 (TAK1) elicits the direct activation of GSDMD by caspase-8 in this context [103, 104]. The knockout or inhibition of caspase-1 triggers the activated AIM2 inflammasome to stimulate caspase-8, leading to caspase-3 activation in macrophages [105, 106]. In the previous section, caspase-8, which is involved in extrinsic apoptotic mechanisms, was reported to promote neuroinflammation after SAH through a nonapoptotic pathway.

Necroptosis is generally considered to be a surrogate owing to its forced response to apoptosis. Thus, an interaction between apoptosis and necroptosis has long been established [107]. The switch molecules in the two death pathways are caspase-8 and its interactor and substrate (RIP1) [108]. In particular, caspase-8 cleaves the RIP1–RIP3 complex upon activation, and in this setting, apoptosis is mediated by activated caspase-8. In contrast, if caspase-8 is inhibited or its activation is disrupted, the complex formed between RIP1 and RIP3 becomes dominant after phosphorylation, leading to necroptosis [109]. Based on a recent study, the switch between necroptosis and apoptosis may also be determined by the amount of RIP1 expression; higher expression of RIP1 leads to necroptosis, whereas inhibition or inactivation of RIP1, which induces a low level or no RIP1, leads to apoptosis [110]. The critical role of necroptosis in EBI after SAH has been previously demonstrated [68]. However, the putative role of RIP3 in converting necroptosis to apoptosis in cells in the brain after SAH has not been determined; thus, follow-up studies are expected. The crucial molecule for its interaction is MLKL, which is activated downstream. Existing evidence suggests that MLKL mediates pyroptosis via two strategies: activating downstream TLRs or TNFR1/2 [111–114] or causing membrane integrity loss after activation, thereby mediating MLKL-dependent potassium efflux [115, 116]. Caspase-8 may be regulated in this process, indicating that some mechanisms of apoptosis activation may occur in necroptosis.

Owing to more research on the interaction of RCD, such as apoptosis, pyroptosis, and necroptosis, whether the three death modes coexist and work together to regulate cell death should be elucidated. No single form of death pathway is sufficient to explain the characteristics caused by this pattern. Karki et al. proposed the concept of “PANoptosis” and conducted a series of studies [117–119]. PANoptosis is a unique RCD that activates the promoter proteins of pyroptosis, apoptosis, and necroptosis through specific pathways involving inflammatory cytokines produced during certain diseases and drives them to assemble inflammasomes specific to different RCD forms [120, 121]. Inflammasomes assemble a complex called the PANoptosome [122], which exacerbates cell death due to preexisting diseases. A relatively complete study of PANoptosis was first published in 2016. PANoptosis was reported to be activated by Z-DNA-binding protein 1 (ZBP1) in the context of viral infection with the influenza A virus (IAV). Therefore, in addition to specific triggers (IAV) and sensors (ZBP1), the PANoptosome assembly of ZBP1 also includes molecules that activate downstream effectors (caspases-1/3/7/8, RIP3, and GSDMs) and downstream executive molecules of each RCD pathway. To date, the domains of PANoptosome components have been divided into three categories: assembly domain, catalytic domain, and sensing domain. The assembly domains include CARD, DEATH, DED, PYD, and RHIM. Four of these assembly domains (CARD, DEATH, DED, and PYD) are also known as death-fold domains owing to their critical role in assembling the RCD executive complex. The catalytic domain is the key domain of downstream molecules, including caspases, RIP1, RIP3, and NLRP3 [122].

Current studies on the PANoptosome are related to infectious diseases and cancer. Accordingly, no studies have been performed on the relation between the PANoptosome and SAH. However, the “raw materials” constituting PANoptosomes in SAH were most highly expressed, suggesting that the search for PANoptosomes has a molecular basis. In the earlier section, the focus was to identify evidence of the RCD crosstalk in SAH; however, PANoptosome formation seems highly trigger-dependent. Thus, future work should focus on identifying specific PANoptosis-inducing sensors that are prominent for the therapeutic understanding of this pathway and determining its modulation and approaches to improve patient outcomes.

## 9. Inhibitors of Caspases in SAH

Numerous clinical studies and experimental data have demonstrated the critical role of caspases in various neurological diseases, implying the foreseeable and essential role of caspases as potential therapeutic targets. As core mediators of apoptosis, caspases are untapped and tractable targets for treating neurodegenerative diseases and brain injury as well as preventative therapeutic approaches [123]. Currently, many natural and synthetic caspase inhibitors have been discovered and developed for therapeutic and research purposes.

The IAP family is a crucial player in some human diseases and a natural inhibitor of caspases. To date, six relatives, including XIAP, BRUCE, NIAP, and survivin, have been identified [124, 125]. In the IAP family, XIAP may be the most effective endogenous apoptosis inhibitor and has high efficiency and affinity for caspase. IAP contains a baculovirus inhibitor of the apoptosis repeat motif at its N-terminus; a C-terminal RING zinc finger domain, which participates in protein-protein and protein-nucleic acid interactions; and a caspase recruitment domain [126]. XIAP can directly inhibit caspase-3 and caspase-9, as well as be affected by the Bax/cytochrome c pathway to inhibit caspase-9 [127]. Thus, XIAP overexpression may have an antiapoptotic effect. Based on recent studies, an increase in XIAP levels protects EBI after SAH. The role of XIAP may be related to the inhibition of the caspase-dependent endogenous apoptosis pathway [128]. The results of current studies suggest that the elevated expression levels of XIAP in SAH may be a protective switch for some type of “safety regulation,” which prevents EBI after SAH [128].

VX-765 is an artificial, reversible caspase-1 inhibitor under development for the treatment of inflammatory diseases [129]. VX-765 attenuates postepileptic-induced IL-1*β* production in the CNS while shortening the duration of epilepsy [130]. The use of VX-765 in a rat model of Alzheimer's has been demonstrated to delay cognitive decline and alleviate cognitive impairment [131]. These results indicate that the therapeutic effect of VX-765 on the CNS has immense potential. In a recent study, VX-765 treatment alleviated the release of neuroinflammatory factors caused by pyroptosis after SAH. It eased the SAH-induced loss of neurons in the hippocampal CA1 region, improving the long-term outcome of SAH [132]. An in-depth study of VX-765 might be a potential and promising direction for EBI treatment after SAH.

N-Ac-Asp-Glu-Val-Asp-CHO (Ac-DEVD-CHO) is a caspase-3 inhibitor, whereas Z-Val-Ala-Asp-fluoromethyl ketone (Z-VAD-FMK) is a broad-spectrum caspase inhibitor with properties of irreversibility and cell permeability that can inhibit inflammation and apoptosis. Both caspase inhibitors have been previously reported to prevent brain endothelial cell apoptosis and reduce vasospasm after SAH, in which Z-VADFMK may act on multiple caspases (such as caspase-3 and caspase-8) to exert its function [30]. In a subsequent study, Z-VAD-FMK reduced IL-1*β* release in SAH-affected rabbit cerebrospinal fluid [133]. Vasospasm was also found to be alleviated by Z-VAD-FMK, which inhibited caspase-1 inactivation and subsequently reduced IL-1*β* production. The prevention of lung endothelial cell apoptosis by Z-VAD-FMK significantly reduced neurogenic pulmonary edema (NPE) [134].

The selective caspase-1 inhibitor, acetyl-tyrosine-valine-alanine-aspartate-chloromethyl ketone (Ac-YVAD-CMK), inhibits the precursor, IL-1*β*, in SAH and attenuates SAH-induced neuronal injury, BBB disruption, and cerebral edema [85, 135]. Ac-YVAD-CMK also prevents NPE from SAH. However, this study also revealed that the drug might activate apoptosis through pathways other than caspase-1 inhibition. As a synthetic caspase-8 inhibitor, Z-IETD-FMK was found to ameliorate neuronal damage after 24 and 72 h of SAH and reduce brain edema. In a follow-up experiment, Z-IETD-FMK was found to improve neural function and motor coordination in SAH mice.

Studies on caspase inhibitors in SAH have revealed their critical status. Nonetheless, to date, no caspase-inhibiting drugs have been used for the clinical treatment of SAH, which is mainly due to side effects, including toxicity and poor pharmacokinetic properties. In addition, caspase inhibition can mediate apoptosis by activating caspase-independent pathways. According to some studies, the actual function of caspases goes far beyond the regulation of cell death and inflammatory responses [136]. Therefore, further animal and preclinical studies are required for the practical clinical application of caspase inhibitors. Reducing drug toxicity and improving target contact specificity may be critical steps in developing caspase-inhibiting drugs.

## 10. Perspectives and Conclusion

SAH is a high-mortality, devastating disease in which EBI harbors a complex, multimechanism-related pathological process. Accordingly, our exploration of its pathological process and underlying mechanisms will continue. Identifying a suitable target in the complicated mechanisms of damage in EBI is of considerable significance for its clinical treatment. In this review, we mainly focused on describing the mechanistic role of caspases in EBI. Caspases have been identified in other neurological diseases [23]. Further, the long-term role of apoptosis and its function in various RCDs have been gradually explored. The experimental and clinical data provided to date suggest that after SAH, caspase is central to the etiology of EBI via the induction of neuronal death. The contribution of caspases to subsequent pathological processes, such as neuroinflammation and BBB destruction, is further highlighted. However, in addition to the known effects of caspases on neuronal, glial, and endothelial cells, we also opted to determine whether caspases mediate EBI through damage to other cells. We demonstrated that the disruption of EBI in SAH is mediated by caspases in specific subcellular organelles, resulting in morphological or functional structural changes that induce RCDs. Based on results obtained by evaluating the mitochondria, ER, and nucleus, caspases may also have undiscovered connections with other organelles, such as ribosomes and Golgi apparatus, which serves as a different research direction and opportunity to better elucidate the complex pathological process of EBI.

During our review, we observed that some apoptotic pathways, such as TNF-*α*-mediated exogenous apoptosis and caspase-independent apoptotic pathways in SAH, were less explored and relatively outdated. Further, caspase-8, which originally mediates the extrinsic apoptosis pathway, is not as simple as expected and is involved in various types of RCDs. Further research on the function and mechanism of caspase-8 will provide a clearer outlook on the processes involved in EBI. It may indicate that the mechanism of exogenous apoptosis is far less simple than previously thought. These studies may clarify whether it is the role of its pathway itself or the effect of crosstalk between different types of RCDs mediated by caspase-8 that engage in EBI. It may reveal hidden directions and promising ideas for further experimental research on EBI. The unexpectedly extensive crosstalk of caspases between RCDs has been demonstrated in other neurological diseases and validated in SAH. Autophagy, a newly discovered form of cell death, has also been identified to involve caspases and plays a crucial role in EBI [137]. Future studies should evaluate multiple forms of cell death in combination rather than individually. The concept of PANoptosis has introduced new notions for studies on EBI. Previously established notions regarding cell death pathways do not conflict with PANoptosis and do not contradict the current understanding of RCDs. Owing to the particularity of PANoptosis, the key research direction should involve the identification of the unique “PANoptosome” of different diseases and the upstream biological factors that specifically induce this mechanism. Obtaining definitive evidence for PANoptosis in SAH, which would enable exciting possibilities for further studying the mechanism of EBI, is anticipated as more information is obtained on the complex regulatory mechanisms of RCDs.

Various synthetic varieties of caspase inhibitors have been discovered recently; however, only a few studies have been performed on SAH. The limited studies may be due to the existence of too many regulatory factors that inhibit the targeting activity of caspase. Further, apoptotic or inflammatory functions may not be straightforward. Caspase inhibitors have potential clinical applications owing to their constantly renewed synthesis process. Furthermore, the newly discovered functions of caspase inhibitors suggest that further exploration of their role in SAH pathology is warranted. Thus, attention should be paid to this fascinating therapeutic target.

In summary, we explored the possible mechanisms and biological functions of caspases in EBI after SAH and highlighted their extensive and complex crosstalk. An improved understanding of the interrelationships between RCDs may enable the development of better and more treatments for SAH. Notably, the study of EBI after SAH, with an in-depth study of caspases, is both an opportunity and a challenge.
